# Ulcerative colitis after SARS-CoV-2 infection

**DOI:** 10.4322/acr.2021.378

**Published:** 2022-04-28

**Authors:** Sofia Kartsoli, Spyridon Vrakas, Dimitrios Kalomoiris, Kassiani Manoloudaki, Vasileios Xourgias

**Affiliations:** 1 Tzaneio General Hospital of Piraeus, Department of Gastroenterology, Piraeus, Greece; 2 Tzaneio General Hospital of Piraeus, 3rd Department of Internal Medicine, Piraeus, Greece; 3 Tzaneio General Hospital of Piraeus, Department of Pathology, Piraeus, Greece

**Keywords:** COVID-19, SARS-CoV-2, Ulcerative colitis

## Abstract

Although severe acute respiratory syndrome coronavirus-2 (SARS-CoV-2) affects mainly the respiratory system, the gastrointestinal tract is also considered a site of viral activity. We hereby present the case of a 74-year-old male patient with the diagnosis of new-onset ulcerative colitis. One month earlier, the patient presented fever, running nose, and diarrhea and was tested positive for SARS-CoV-2. Studies with COVID-19 patients revealed significant changes in gut microbiota composition and alterations in immune responses that could lead to chronic inflammation and manifestations of inflammatory bowel disease. We review additional cases of ulcerative colitis presented after SARS-CoV-2 infection and summarize the possible mechanisms that underlie the gastrointestinal abnormalities in COVID-19 patients.

## INTRODUCTION

Severe acute respiratory syndrome coronavirus 2 (SARS-CoV-2), a novel coronavirus first emerged in Wuhan, China is the etiological agent of Coronavirus Disease 2019 (COVID-19).^1^ Although pulmonary symptoms and respiratory disorders are considered the most common manifestations of COVID-19, up to 20% of infected patients have reported gastrointestinal (GI) symptoms, such as diarrhea, nausea, vomiting, and abdominal pain.^2^ The consequences of gastrointestinal disorders during COVID-19 are under investigation, and the potential long-term effects of intestinal inflammation remain unclear.

Ulcerative colitis (UC) is a prevalent chronic inflammatory condition of the colon. It is characterized by continuous mucosal inflammation, resulting in friability and superficial erosions of the colonic wall. The disease is believed to arise in genetically susceptible individuals, and at the same time environmental factors, autoimmunity, and gut microbiota are considered to be pivotal factors in the pathogenesis of UC.^3^^,^^4^ Actually, bacterial infections and viral gastroenteritis have been implicated in the development and pathophysiology of inflammatory bowel disease (IBD).^5^^,^^6^

We report the case of a man who was diagnosed with ulcerative colitis one month after COVID-19 infection and discuss the possible pathogenetic role of SARS-CoV-2 in triggering chronic inflammation.

## CASE REPORT

A 74-year-old nonsmoking Caucasian male patient sought medical care complaining of fever (38^o^C), running nose, and watery diarrhea over the last 5 days. He denied abdominal pain. His medical history was non-contributive. He tested positive for the novel coronavirus by RT-PCR test; however, his clinical presentation did not require hospitalization and was managed with self-isolation at home for 14 days. Running nose and fever subsided after 5 days, and diarrhea after 15 days of the diagnosis. The patient did not take non-steroidal anti-inflammatory drugs and had no history of gastrointestinal symptoms before the SARS-CoV-2 infection.

One month later, the patient presented to the emergency department with a 7-day history of fever up to 38^o^C and bloody diarrhea (5 stools per day). Laboratory analyses revealed anemia, Hb was 9g/dl (RR: 13.5-17.5 g/dL) with normal MCV and MCH, elevated C-reactive protein (20,3mg/L, RR: <3,14mg/L)), platelet count 450 × 10^9^/L (RR: 150-400 × 10^9^/L), serum albumin 4.2 g/dL (RR: 3.5-5 g/dL), while other laboratory results and thoracic radiograph were unremarkable. Nasopharyngeal swab for SARS-CoV-2 was negative, and the patient was admitted for further evaluation and treatment. Stool studies, including cultures for common pathogens, parasites, tests for *Clostridium difficile* GDH, toxins A and B all returned negative. Moreover, the gastrointestinal PCR panel test for common pathogens was also negative. This panel test targets bacterial infections (*Campylobacter jejuni*, *Campylobacter coli*, *C. difficile* toxin A/B, *Salmonella* spp, *Vibrio cholerae*, *Yersinia enterocolitica*, Enteroaggregative *E. coli*, Enteropathogenic *E. coli*, Enterotoxigenic *E. coli*, Shigella-like toxin-producing *E. coli*, *Shigella* spp), parasitic infections (Cryptosporidium, *Entamoeba histolytica*, *Giardia lamblia*) and viral infections (Adenovirus, Astrovirus, Norovirus, Rotavirus A, Sapovirus).

**Figure 1:**
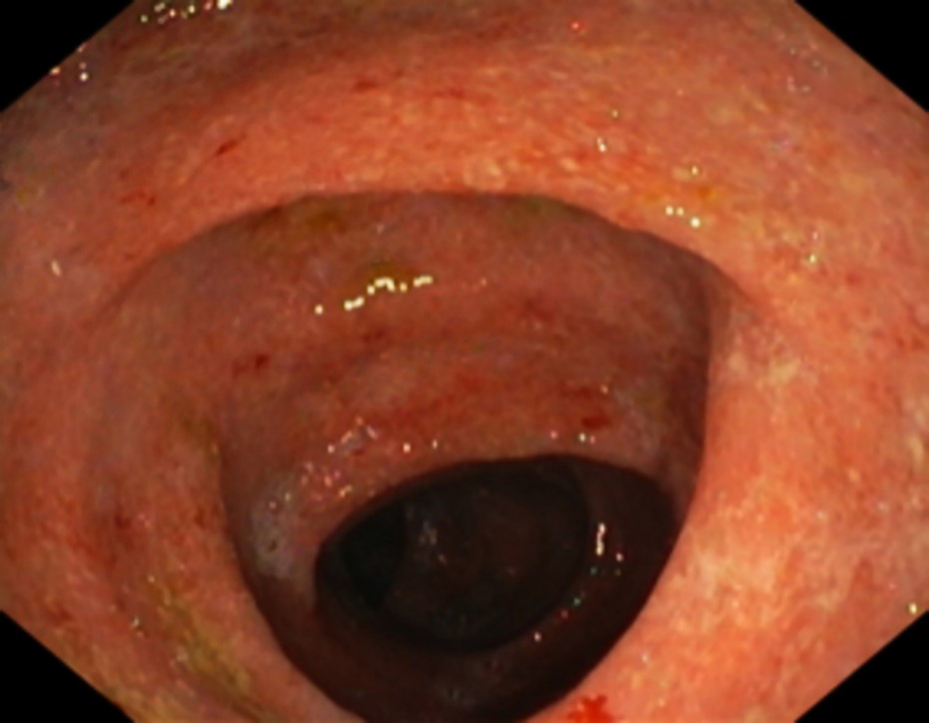
Colonoscopic view of the mucosal inflammation of the descending colon with mucosal friability, edematous mucosa, erythema and loss of vascular markings.

Ileocolonoscopy showed continuous mucosal inflammation extending from the rectum to ascending colon with mucosal friability, edematous mucosa, erythema and loss of vascular markings (Figure 1).

**Figure 2:**
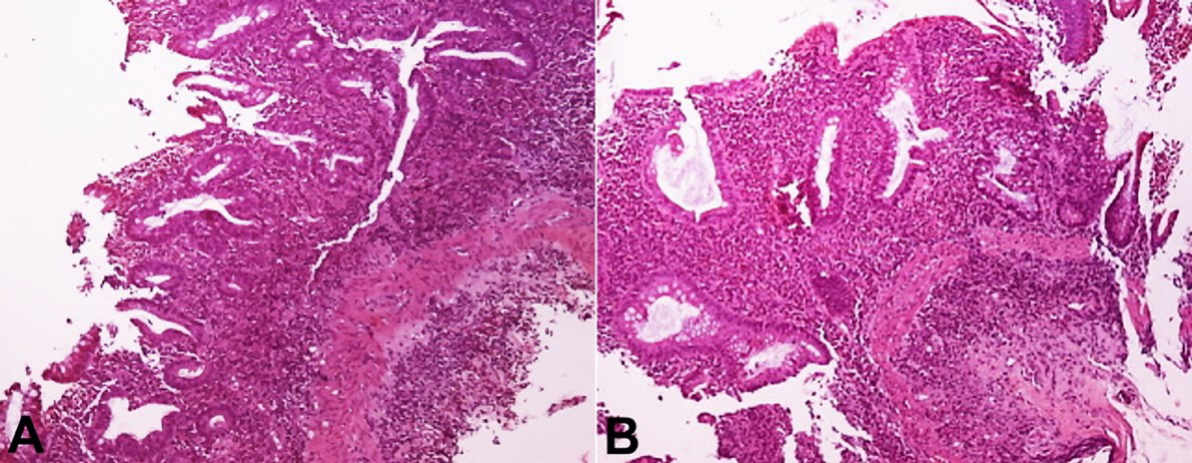
Photomicrographs of the colonic mucosal biopsy indicating ulcerative colitis. **A** and **B** – Crypt architectural distortion- basal plasmacytosis (H&E, x10) (H&E, x20).

**Figure 3:**
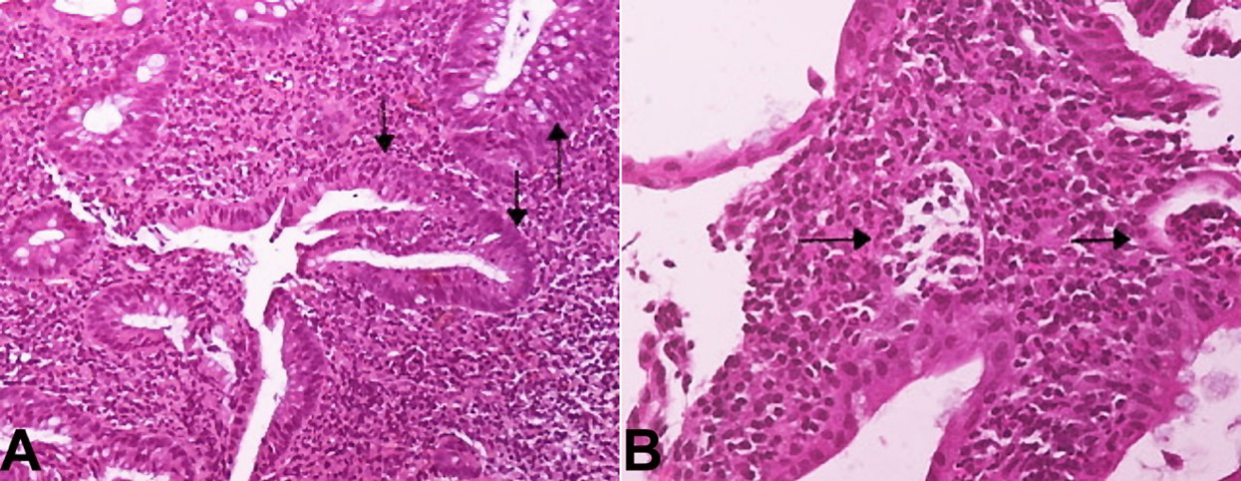
Photomicrographs of the colonic mucosal biopsy indicating ulcerative colitis. **A** – Cryptitis (H&E, x20); **B –** Crypt abscesses (H&E, x40).

Mucosal of terminal ileum and cecum was normal. Biopsies were taken from mucosal lesions for histopathology examination and RT-PCR test for SARS-CoV-2. Colonic biopsy samples were negative for SARS-CoV-2 and Cytomegalovirus (CMV) inclusions. Hematoxylin and eosin staining showed basal plasmacytosis, superficial erosions, crypt architectural distortion (Figure 2A, and 2B), mucin depletion, a diffuse and active inflammatory infiltrate with cryptitis and crypt abscesses (Figure 3A and 3B).

A diagnosis of ulcerative colitis was made, and treatment with oral and topical mesalamine was started. After a 15-day treatment with per os and per rectum mesalamine, complete resolution of the patient’s symptoms was achieved. The patient remained in clinical remission with mesalamine and underwent a colonoscopy 6 months after treatment, which showed mucosal healing.

## DISCUSSION

The ongoing COVID-19 pandemic has unveiled many clinical challenges because of its unpredictable disease kinetics. Since the outbreak of COVID-19, 4 cases of ulcerative colitis following SARS-CoV-2 infection have been reported (two cases in young females and two cases in male patients aged 50 and 55 years old).^7^^-^10 SARS-CoV-2 infection in the aforementioned patients was confirmed with a positive nasopharyngeal swab, except for the 50-year-old patient who was considered to have COVID-19 pneumonia because of CT findings compatible with viral pneumonia and positive RT-PCR tests of his family.^10^ Moreover, COVID-19 pneumonia was diagnosed in three of the four cases, while one patient presented only anosmia and ageusia, fever, and gastrointestinal symptoms such as nausea, vomiting, and bloody diarrhea.^9^ All patients received treatment for COVID-19 and required hospitalization.

It is worth mentioning that all cases were reported during the first wave of COVID-19 in Europe. At that time, little was known about the clinical management of patients with COVID-19, and proven specific therapies were unavailable. Hydroxychloroquine has been given as off-label therapy for COVID-19, and three out of four patients received it as monotherapy or in combination with antimicrobial agents, such as azithromycin and lopinavir/ritonavir.^11^ Conversely, a patient received steroids and azithromycin.^8^

Another interesting point is the time interval between SARS-CoV-2 infection and the manifestations and diagnosis of ulcerative colitis observed in these cases. In three cases, ulcerative colitis symptoms presented shortly after completion of treatment for COVID-19, while the patient who received steroids for COVID-19 pneumonia presented diarrhea and was diagnosed with UC 3 months after SARS-CoV-2 infection.^7^^-^10 In our case, the patient was 74 years old with no history of gastrointestinal symptoms before the SARS-CoV-2 infection. The patient presented bloody diarrhea and fever one month after the SARS-COV-2 infection. COVID-19 was ruled out by negative nasopharyngeal swab and by negative biopsy samples. Therefore endoscopic and histologic findings are more likely attributed to ulcerative colitis.

Gastrointestinal disorders and symptoms in COVID-19 patients have been reported by several epidemiological research groups during the early months of pandemic.^2^ However, the etiological factors for these disorders remain under investigation. SARS-CoV-2 RNA has been detected in stool samples, anal swabs, and gastrointestinal histological samples based on RT-PCR in infected patients, suggesting that the gut is a possible site of viral replication and activity.^2^ ACE-2 is highly expressed in enterocytes and colonocytes and SARS-CoV-2 presents an elevated binding affinity to these receptors.^4^ Moreover, fecal calprotectin, a marker of inflammatory responses in the gut, was found to be elevated in these patients with diarrhea, implicating that SARS-CoV-2 infection triggers inflammation and cell injury in the digestive tract.^12^ Viral replication, cellular damage and the subsequent viral secretions might directly induce injury and inflammation of the gut epithelium and dysfunction of the intestinal barrier.^13^ In fact, intestinal infection with SARS-like viruses has been reported to lead to overexpression of several enzymes in the atypical surface of mature enterocytes altering and possibly disrupting their function and eventually causing cellular damage and enterocytes apoptosis.^14^ Furthermore, viral secretions lead to the release of cytokines and inflammatory responses.^13^ Indeed, a large number of T-lymphocytes and mononuclear macrophages were found to be in activated state in COVID-19 patients, resulting in expression and release of cytokines.^15^ Moreover, in these patients, the levels of cytokines, and specifically those of interleukin-6 and interleukin-23, are found to be increased.^16^^,^^17^ The inflammatory responses described above are involved in inflammatory pathways and pathogenesis of ulcerative colitis, and a possible exaggeration of immune response with the impact of additional factors could induce ultimately chronic colonic inflammation in certain patients with COVID-19.^18^

ACE-2 is considered to be a key regulator of intestinal homeostasis, intestinal microflora, and immunity. Mice lacking ACE-2 present significant alterations in gut microbiota composition, which is mainly attributed to the reduced production of antimicrobial peptides.^19^ During SARS-CoV-2 infection, the interaction between virus and ACE-2 receptor can lead to decreased availability of the latter or even deregulation, resulting in several changes in the gut microbiota composition. It is well known that gut microbiota plays an important role in host physiology and changes in its composition have numerous effects on immune response and metabolism.^20^ In fact, pilot studies have demonstrated significant alterations in intestinal microbiomes of patients with SARS -CoV-2 infection, even in patients naïve to antibiotic therapy.^21^ In fecal samples of infected patients there was significant depletion of bacterial diversity and bacterial abundance. Opportunistic bacterial pathogens were also enriched in fecal samples of patients with COVID-19, while beneficial commensals were found in relatively lower abundance.^21^^,^^22^ It is noteworthy that the depletion of beneficial bacteria persisted in most COVID-19 patients even after the clearance SARS-CoV-2, indicating that infection may be associated with a long-lasting impaired gut microbiome with defective function.^21^ Bacteroidetes species were found in low abundance in patients with COVID-19, and specific species, among them *B. dorei,* were negatively correlated with a viral load of SARS-CoV-2.^21^ These species have been reported to downregulate ACE-2 expression, and it is plausible to assume that these alterations could influence immune responses.^21^ Moreover, the depletion of beneficial commensals, such as an anti-inflammatory bacterium, observed in these patients accompanied by overgrowth of opportunistic pathogens may cause damage to the intestinal mucosal barrier, the release of inflammatory cytokines and eventually induce colonic inflammation. Interestingly, intestinal microbiota dysbiosis is considered a major contributing factor in the pathogenesis of ulcerative colitis.^4^ Low bacterial diversity was also reported in UC patients.^4^^,^^23^ Furthermore, the number of pathogens, as in SARS-CoV-2 infection, is found to be increased in patients with ulcerative colitis.^22^ These alterations and the instability of gut microbiota are considered to be major contributing factors in the pathogenesis of UC. Hence, SARS- CoV-2 infection and the subsequent altered intestinal microbiota could initiate abnormalities involved in the development of ulcerative colitis.

## CONCLUSION

In addition to the pulmonary tract, SARS-CoV-2 infection also seem to affect the gastrointestinal tract significantly. Local immune deregulation and fecal microbiota disturbances followed by COVID-19 could induce chronic colonic inflammation and eventually lead to the development of ulcerative colitis. It is important to note that causation cannot be proved, but an association is plausible. Further prospective studies in patients who recovered from COVID-19, focusing mainly on symptoms and diseases of the gastrointestinal tract, would help to unravel the long-term impacts of SARS-CoV-2 infection.

## References

[1] Zhu N, Zhang D, Wang W (2020). A novel coronavirus from patients with pneumonia in China, 2019. N Engl J Med.

[2] Cheung KS, Hung IFN, Chan PPY (2020). Gastrointestinal manifestations of SARS-CoV-2 infection and virus load in fecal samples from a hong kong cohort: systematic review and meta-analysis. Gastroenterology.

[3] Porter RJ, Kalla R, Ho G-T (2020). Ulcerative colitis: recent advances in the understanding of disease pathogenesis. F1000Res.

[4] Shen Z-H, Zhu C-X, Quan Y-S (2018). Relationship between intestinal microbiota and ulcerative colitis: mechanisms and clinical application of probiotics and fecal microbiota transplantation. World J Gastroenterol.

[5] Sun L, Nava GM, Stappenbeck TS (2011). Host genetic susceptibility, dysbiosis, and viral triggers in inflammatory bowel disease. Curr Opin Gastroenterol.

[6] García Rodríguez LA, Ruigómez A, Panés J (2006). Acute gastroenteritis is followed by an increased risk of inflammatory bowel disease. Gastroenterology.

[7] Taxonera C, Fisac J, Alba C (2021). Can COVID-19 trigger de novo inflammatory bowel disease?. Gastroenterology.

[8] Imperatore N, Bennato R, D’Avino A, Lombardi G, Manguso F (2021). SARS-CoV-2 as a trigger for de novo ulcerative colitis. Inflamm Bowel Dis.

[9] Calabrese E, Zorzi F, Monteleone G, Del Vecchio Blanco G (2020). Onset of ulcerative colitis during SARS-CoV-2 infection. Dig Liver Dis.

[10] Aydın MF, Taşdemir H (2021). Ulcerative colitis in a COVID-19 patient: a case report. Turk J Gastroenterol.

[11] Kalil AC (2020). Treating COVID-19: off-label drug use, compassionate use, and randomized clinical trials during pandemics. JAMA.

[12] Effenberger M, Grabherr F, Mayr L (2020). Faecal calprotectin indicates intestinal inflammation in COVID-19. Gut.

[13] Dahiya DS, Kichloo A, Albosta M, Pagad S, Wani F (2020). Gastrointestinal implications in COVID-19. J Investig Med.

[14] Ding S, Liang TJ (2020). Is SARS-CoV-2 also an enteric pathogen with potential fecal-oral transmission? A COVID-19 virological and clinical review. Gastroenterology.

[15] Xu X, Han M, Li T (2020). Effective treatment of severe COVID-19 patients with tocilizumab. Proc Natl Acad Sci USA.

[16] Zhang Y, Li J, Zhan Y (2004). Analysis of serum cytokines in patients with severe acute respiratory syndrome. Infect Immun.

[17] Sadeghi A, Tahmasebi S, Mahmood A (2021). Th17 and Treg cells function in SARS-CoV2 patients compared with healthy controls. J Cell Physiol.

[18] Dvornikova KA, Bystrova EY, Churilov LP, Lerner A (2021). Pathogenesis of the inflammatory bowel disease in context of SARS-COV-2 infection. Mol Biol Rep.

[19] Hashimoto T, Perlot T, Rehman A (2012). ACE-2 links amino acid malnutrition to microbial ecology and intestinal inflammation. Nature.

[20] Guinane CM, Cotter PD (2013). Role of the gut microbiota in health and chronic gastrointestinal disease: understanding a hidden metabolic organ. Therap Adv Gastroenterol.

[21] Zuo T, Zhang F, Lui GCY (2020). Alterations in gut microbiota of patients with COVID-19 during time of hospitalization. Gastroenterology.

[22] Gu S, Chen Y, Wu Z (2020). Alterations of the gut microbiota in patients with coronavirus disease 2019 or H1N1 influenza. Clin Infect Dis.

[23] Alipour M, Zaidi D, Valcheva R (2016). Mucosal barrier depletion and loss of bacterial diversity are primary abnormalities in paediatric ulcerative colitis. J Crohn’s Colitis.

